# Purifying selection in mitochondria, free-living and obligate intracellular proteobacteria

**DOI:** 10.1186/1471-2148-7-17

**Published:** 2007-02-12

**Authors:** Leila Mamirova, Konstantin Popadin, Mikhail S Gelfand

**Affiliations:** 1 Institute for Information Transmission Problems RAS, Bolshoi Karetny pereulok 19, Moscow 127994, Russia; 2 Department of Genetics, Biological Faculty of M.V. Lomonosov Moscow State University, Vorobyevy Gory 1-12, Moscow 119992, Russia; 3 Faculty of Bioengineering and Bioinformatics, M.V. Lomonosov Moscow State University, Vorobyevy Gory 1-73, Moscow 119992, Russia

## Abstract

**Background:**

The effectiveness of elimination of slightly deleterious mutations depends mainly on drift and recombination frequency. Here we analyze the influence of these two factors on the strength of the purifying selection in mitochondrial and proteobacterial orthologous genes taking into account the differences in the organism lifestyles.

**Results:**

(I) We found that the probability of fixation of nonsynonymous substitutions (*K*_*n*_*/K*_*s*_) in mitochondria is significantly lower compared to obligate intracellular bacteria and even marginally significantly lower compared to free-living bacteria. The comparison of bacteria of different lifestyles demonstrates more effective elimination of slightly deleterious mutations in (II) free-living bacteria as compared to obligate intracellular species and in (III) obligate intracellular parasites as compared to obligate intracellular symbionts. (IV) Finally, we observed that the level of the purifying selection (*i.e*. 1-*K*_*n*_*/K*_*s*_) increases with the density of mobile elements in bacterial genomes.

**Conclusion:**

This study shows that the comparison of patterns of molecular evolution of orthologous genes between ecologically different groups of organisms allow to elucidate the genetic consequences of their various lifestyles. Comparing the strength of the purifying selection among proteobacteria with different lifestyles we obtained results, which are in concordance with theoretical expectations: (II) low effective population size and level of recombination in obligate intracellular proteobacteria lead to less effective elimination of mutations compared to free-living relatives; (III) rare horizontal transmissions, i.e. effectively zero recombination level in symbiotic obligate intracellular bacteria leads to less effective purifying selection than in parasitic obligate intracellular bacteria; (IV) the increased frequency of recombination in bacterial genomes with high mobile element density leads to a more effective elimination of slightly deleterious mutations. At the same time, (I) more effective purifying selection in relatively small populations of nonrecombining mitochondria as compared to large populations of recombining proteobacteria was unexpected. We hypothesize that additional features such as the high number of protein-protein interactions or female germ-cell atresia increase evolutionary constraints and maintain the effective purifying selection in mitochondria, but more work is needed to definitely establish these additional features.

## Background

Theoretically, elimination of recent slightly deleterious mutations must be more effective in species with high population size [1,2], since low stochastic sampling variance decreases the power of random genetic drift. Also, according to the theory, meiotic segregation and recombination allow for independent mixing of alleles, thus leading to more effective elimination of slightly deleterious mutations [3-6] and halting of the Muller's ratchet [7,8]. Thus both the population size and the recombination level determine the effectiveness of the purifying selection.

Although the influences of population size and recombination level on the effectiveness of the purifying selection are interconnected and often mutually reinforcing, it is possible to distinguish between them by comparing species with different population sizes, levels of recombination, or both. For example, the influence of the population size on probability of fixation of slightly deleterious mutations was observed in comparison of primates and rodents [9-11], mammals, birds, and drosophilids [12], island- versus continent-inhabiting populations of same species [13], and large- versus small-bodied mammals[14]. The role of the recombination level was demonstrated in asexual versus sexual *Daphnia *[15]. The influence of both population size and recombination level, most likely, leads to the differences in the molecular evolution patterns of endosymbiotic and free-living bacteria [16-18] and to degeneration of the neo-Y chromosome of *Drosophila miranda *[19].

The strength of the purifying selection, acting on different genes, is mainly determined by structural or functional constraints on the encoded protein. Since orthologous proteins in closely related species most likely operate under similar constraints, it is possible to investigate more intimate relationships between the rates of molecular evolution, the population sizes of the analyzed species and the level of recombination in their genomes. Here we perform a comprehensive analysis of purifying selection in various groups of proteobacteria and mammalian mitochondria.

The relative effectiveness of the purifying selection was estimated as the rate of fixation of nonsynonymous substitutions (*K*_*n*_*/K*_*s*_) and radical amino acid substitutions (*K*_*r*_*/K*_*c*_) in seven orthologous genes, encoding subunits of a respiratory chain enzyme (NADH:ubiquinone-oxidoreductase or complex I) from bacterial and mitochondrial genomes. These genes encode components of one multi-subunit complex and represent a large fraction of all mitochondrial protein-coding genes. Orthologous genes, encoding subunits of complex I, exist in all aerobic bacteria.

Since the respiratory chain proteins are highly conserved, we assume that the role of the positive selection is negligible, and thus these genes are appropriate for the analysis of the purifying selection. Throughout the work we empirically corroborate this assumption.

*Firstly*, we compare mammalian mitochondria with bacteria. Since mitochondria are descendants of ancient endosymbiotic proteobacteria, it is interesting to compare the purifying selection in orthologous mitochondrial and proteobacterial genes with regards to the differences of their ecology. Mitochondrial genes of vertebrates are strictly maternally inherited, and thus asexual, and have low population size [20]. Conversely, the bacteria (especially free-living) are characterized by nonzero recombination levels and high population sizes. Therefore, we can expect that mitochondria are subject to less effective purifying selection than bacteria.

*Secondly*, we consider the purifying selection in various groups of proteobacteria, taking into account their ecology. From the ecological point of view, the analyzed sample of bacterial species consists of two main groups: obligate intracellular species (parasites or mutualists) and free-living species able to exist outside of the host cell. The free-living protebacteria are characterized by higher population size and higher recombination level, and thus one may expect that they are subject to more effective purifying selection as compared to obligate intracellular species [21,18].

*Thirdly*, we compare the purifying selection in obligate intracellular symbionts (gamma-proteobacteria) and obligate intracellular parasites (alpha-proteobacteria). It is generally assumed that among obligate intracellular bacteria, symbionts with strictly vertical inheritance (such as obligate dietary endosymbiotic gamma-proteobacteria: *Buchnera *and *Blochmannia*, that are required for the survival and reproduction of their insect host) possess lower level of recombination as compared with parasites (such as some alpha-proteobacteria: human pathogen *Rickettsia *and reproductive parasite of arthropods *Wolbachia*) which are able to switch from one host to another and thus contact and recombine with a novel gene pool [22]. Thus we test the hypothesis that the transmission differences among obligate intracellular species, determined by the differences in their ecology, might influence the effectiveness of purifying selection through recombination frequency.

*Fourthly*, we analyze the correlation of the strength of purifying selection with the mobile elements density in bacterial genomes. We expect that the mobile element density in bacteria should positively correlate with the recombination level, as it seems likely that these are two reciprocally reinforcing factors. The inter-genomic recombination level in bacteria is mainly determined by the activity of the mobile elements which able to perform conjugative transfer (conjugative plasmids, conjugative elements) or transduction (prophages of temperate phages) [23,24]. Number of other mobile elements, without ability to induce recombination, is evolutionary maintained in concordance with the recombination level in bacterial genomes [22] as it is in eukaryotes [25-33]. Thus we use the density of all mobile elements as a proxy measure of the recombination activity in bacterial genomes.

Unexpectedly, the level of purifying selection in mitochondria is significantly higher as compared with obligate intracellular bacteria and even marginally significantly higher as compared with free-living bacteria. This observation contradicts the widely accepted opinion about degradation of mitochondrial genomes and we discuss some factors, which can increase selection constraints of mitochondrial genes. The comparison of bacteria demonstrates a more effective elimination of mutations in free-living species as compared to obligate intracellular ones with low effective population size and level of recombination. The purifying selection in parasitic obligate intracellular bacteria is more effective as compared to symbiotic ones, possibly due to the higher recombination level in the former. To elucidate the purifying effect of recombination, we demonstrate that the effectiveness of purifying selection (1-*K*_*n*_*/K*_*s*_) positively correlates with the density of mobile genes, i.e. with the frequency of recombination.

## Results

### (I) Evolutionary models

**Figure 1:**
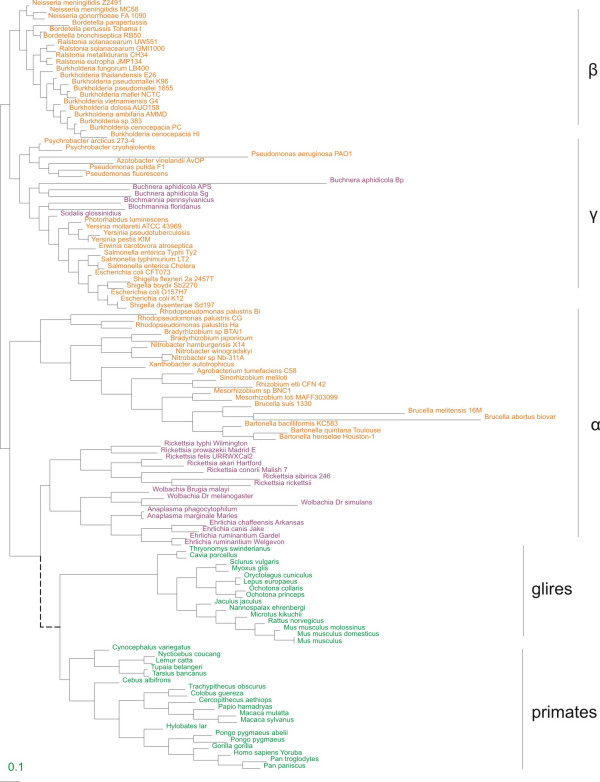
**The phylogenetic tree of the analyzed species**. Green – mammals, purple – obligate intracellular bacteria (endosymbionts in gamma- and parasites in alpha-proteobacteria), orange – free-living bacteria. The branch lengths represent the *K*_*n*_*/K*_*s *_values obtained in model 2c (*K*_*n*_*/K*_*s *_of each external branch were estimated independently, while *K*_*n*_*/K*_*s *_of internal branches were estimated as a constant for each sub-tree.) The dotted line reflects the hypothetical origin of mitochondria from an ancestor common with Rickettsiales.

The phylogenetic tree, used in our analysis, is shown on Fig. 1. To estimate the *K*_*n*_*/K*_*s *_values several models were implemented for the mammalian and bacterial trees: model 0 with a single *K*_*n*_*/K*_*s *_value for all branches of the tree; model 2a with two *K*_*n*_*/K*_*s *_values estimated separately for all external and all internal branches; and model 2b with *K*_*n*_*/K*_*s *_values estimated separately for groups of external branches and a single *K*_*n*_*/K*_*s *_value for all internal branches. In model 2b, bacterial external branches were grouped by ecology or by taxonomy and ecology, mammalian external branches were grouped only by taxonomy.

Further the bacterial tree was divided into 3 monophyletic sub-trees (alpha-, beta and gamma-proteobacteria), and the mammalian tree was divided into glires and primates sub-trees, and each sub-tree was analyzed separately. For each sub-tree three models were implemented: models 0 and 2a defined as above, and model 2c with *K*_*n*_*/K*_*s *_estimated separately for each external branch and a single *K*_*n*_*/K*_*s *_for all internal branches.

Among any two compared nested models, the one with more degrees of freedom had a significantly higher maximum likelihood (ML) value (i.e. 2Δln*L *was larger with statistical significance of at least a 5% for a χ^2 ^distribution with the number of the degrees of freedom dependent on the model), thus providing a better fit to the analyzed data. For the complete bacterial and mammalian trees: ML (models 2b) > ML (model 2a) > ML (model 0); for sub-trees: ML (models 2c) > ML (models 2a) > ML (model 0).

Since in model 2c the external branches of seven species had no nonsynonymous substitutions (*K*_*n *_= 0 and thus *K*_*n*_*/K*_*s *_= 0), these species were not considered further. Therefore, our final dataset for this model consisted of 110 species, including 36 alpha-proteobacteria (20 free-living and 16 obligate intracellular parasites), 20 free-living beta-proteobacteria, 22 gamma-proteobacteria (16 free-living and 6 obligate intracellular symbionts), 13 glires and 19 primates.

**Figure 2:**
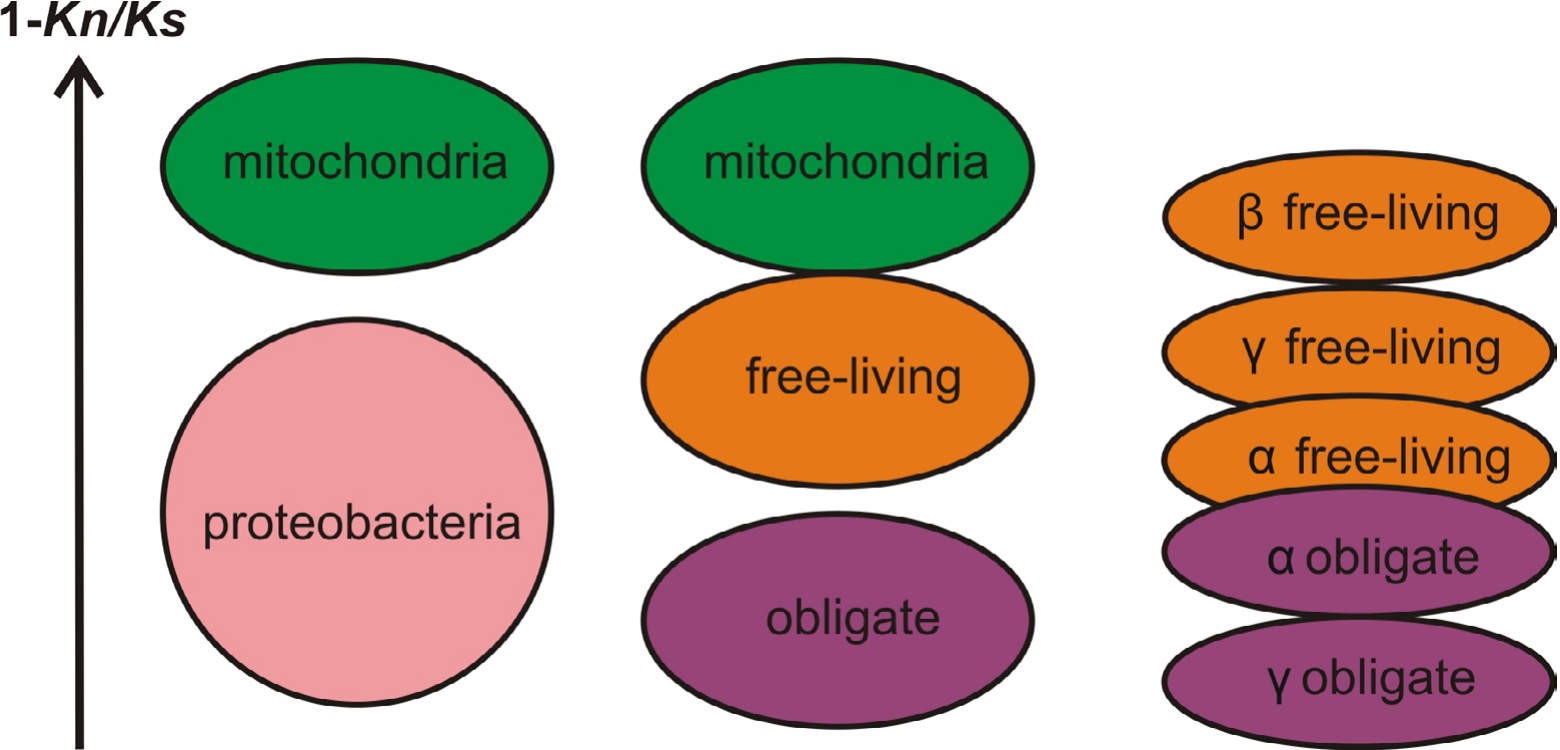
**Significance of the difference of the purifying selection (1-*K*_*n*_*/K*_*s*_) in the analyzed organisms from model 2c**. For ovals within each column, P < 0.05 if ovals are separated; 0.05<P≤0.08 if ovals touch; P > 0.08 if ovals overlap. The more pronounced trend with non-overlapping standard errors is observed in results of models 2a and 2b (see Table 1).

All results obtained from models 2a and 2b (Table 1) were consistent with the results of model 2c (Table 1, Fig. 2), which we will discuss in detail below.

**Table 1:** Means and standard errors of the *K*_*n*_/*K*_*s *_values obtained in models 2a, 2b and 2c.

2a, 2c	2b, 2c	
mammals	glires 0.043 ± 0.0021, 0.049 ± 0.0042	
0.070 ± 0.0017, 0.073 ± 0.0065	primates 0.088 ± 0.0025, 0.089 ± 0.0087	
proteobacteria	free-living 0.102 ± 0.0022, 0.148 ± 0.0303	beta 0.081 ± 0.0038, 0.048 ± 0.0095
0.148 ± 0.0026), 0.192 ± 0.0300		alpha 0.121 ± 0.0036, 0.243 ± 0.0610
		gamma 0.089 ± 0.0038, 0.153 ± 0.0643
	
	obligate 0.305 ± 0.0087, 0.304 ± 0.0689	alpha 0.244 ± 0.0088, 0.224 ± 0.0503
		gamma 0.445 ± 0.0203, 0.519 ± 0.2004

### (II) Lack of positive selection

For all phylogenetic groups (all mammals, primates, glires, all proteobacteria, α-, β-, γ-proteobacteria) model 7 (*K*_*n*_*/K*_*s *_values assumed to be beta-distributed on interval (0, 1)) had significantly lower maximum likelihood (ML) value than model 8 (one extra class of sites which may have *K*_*n*_*/K*_*s *_values larger than one). However, the *K*_*n*_*/K*_*s *_values of the extra class sites were less then one in all mammals (0.298) and in all proteobacteria (0.384), as well as in each group separately: 0.319 in primates, 0.223 in glires, 0.513 in α-, 0.239 in β- and 0.462 in γ-proteobacteria. Thus we did not observe positive selection in the analyzed genes.

### (III) Comparison of mitochondria with bacteria

The *K*_*n*_*/K*_*s *_values of mitochondrial genes are significantly lower than those for all proteobacteria (P = 0.013), as well as for all obligate proteobacteria (P < 0.001) and obligate alpha-proteobacteria (P < 0.001), but only marginally significantly lower than those for all free-living proteobacteria (P = 0.068; Fig. 2). The primate *K*_*n*_*/K*_*s *_values are larger than the glires ones (P < 0.001) and lower than *K*_*n*_*/K*_*s *_for obligate alpha-proteobacteria (P = 0.007). Average values of *K*_*n *_and *K*_*s *_estimated separately for each group are given in Table in additional file 1.

### (IV) Bacterial ecology

In the analysis of all proteobacteria, the obligate intracellular proteobacteria have higher *K*_*n*_*/K*_*s*_, as compared with the free-living ones (P = 0.018; Fig. 2). For gamma-proteobacteria, the obligate intracellular ones have higher *K*_*n*_*/K*_*s *_than the free-living ones (P = 0.032), while for alpha-proteobacteria the obligate intracellular have *K*_*n*_*/K*_*s *_similar to that of free-living ones (P = 0.815; Fig. 2).

The *K*_*n*_*/K*_*s *_values of obligate intracellular alpha-proteobacteria (parasitic) are smaller than those of obligate intracellular gamma-proteobacteria (symbiotic) (Fig. 2) at a marginally significant level (P = 0.054). The free-living alpha-proteobacteria have higher *K*_*n*_*/K*_*s *_values as compared to free-living beta-proteobacteria (P = 0.003), and similar to those of free-living gamma-proteobacteria (P = 0.319; Fig. 2). The *K*_*n*_*/K*_*s *_values for free-living gamma-proteobacteria are marginally higher than for beta-proteobacteria (P = 0.080; Fig. 2). Averages values of *K*_*n *_and *K*_*s *_estimated separately for each group are given in additional file 1.

### (V) Fixation of radical nonsynonymous substitutions (K_r_/K_c_)

Two of the four *K*_*r*_*/K*_*c *_ratios (charge-based *K*_*r*_*/K*_*c *_and volume-based *K*_*r*_*/K*_*c*_) demonstrate significantly higher values in all bacteria compared with all mitochondria (P < 0.001 and P = 0.049, respectively). None of the *K*_*r*_*/K*_*c *_ratios demonstrate significant difference between free-living and obligate intracellular bacteria.

### (VI) Influence of branch lengths

Biased estimations of *K*_*n*_*/K*_*s *_values could be due to the saturation of synonymous sites on long branches [34], or to the stochastic errors on short branches [35]. Since one substitution per codon (i. e. branch length = 1) is the optimal divergence level for the estimation of *K*_*n*_*/K*_*s *_[36] we repeated all analyses using species with the length of the external branch from the interval (0.02, 2). Despite the decreased sample size, the majority of all previously obtained significant results still remained significant. However, some of marginally significant results disappeared (see Table 2).

**Table 2:** Pairwise group comparisons of *K*_*n*_*/K*_*s *_values (estimated in model 2c) for all analyzed species (column "all branches") and for species with external branches of the optimal length (column "optimal branches"). In each comparison, the first mentioned group (before slash) has lower *K*_*n*_*/K*_*s*_ average value compared to the second group (after slash). The significance of differences of the averages was estimated using the t-test. The number of species from both groups is in the brackets, respectively. See the text for details.

compared groups	all branches	optimal branches
mammals/proteobacteria	**P = 0.013 (32/80)**	**P = 0.032 (28/59)**
mammals/obligate proteobacteria	**P < 0.001 (32/22)**	**P < 0.001 (28/15)**
mammals/obligate alpha-proteobacteria	**P < 0.001 (32/16)**	**P < 0.001 (28/9)**
mammals/free-living proteobacteria	P = 0.068 (32/56)	P = 0.171 (28/44)
glires/primates	**P < 0.001 (13/19)**	**P < 0.001 (12/16)**

free-living proteobacteria/obligate proteobacteria	**P = 0.018 (56/22)**	**P = 0.002 (44/15)**
free-living gamma-proteobacteria/obligate gama-proteobacteria	**P = 0.032 (16/6)**	P = 0.059 (13/6)
free-living alpha-proteobacteria/obligate alpha-proteobacteria	P = 0.815 (20/16)	P = 0.163 (17/9)
obligate alpha-proteobacteria/obligate gamma-proteobacteria	P = 0.054 (16/6)	P = 0.165 (9/6)

### (VII) Density of mobile genes

**Figure 3:**
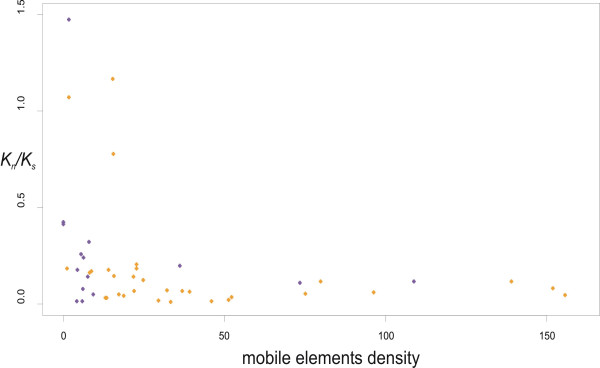
**Relationship of *K*_*n*_/*K*_*s *_and mobile elements density for 46 bacterial genomes**. Mobile elements density was estimated as the number of mobile elements per megabase of bacterial genome. 31 species of free-living bacteria (orange circles) and 15 species of obligate intracellular bacteria (purple circles).

The *K*_*n*_*/K*_*s *_values were negatively correlated with the mobile elements density (hereafter MED: plasmid, prophage and transposable element genes per one megabase of genome size; Kendall tau = -0.305, P = 0.0014, N = 46) (Fig. 3). After exclusion of two species with no mobile elements (*Buchnera aphidicola APS *and *Buchnera aphidicola Sg*), we performed linear regression of Ln(MED) on Ln(*K*_*n*_*/K*_*s*_) that corroborated the observed trend (Ln(*K*_*n*_*/K*_*s*_) = -1.297 - 0.363×Ln(MED), r^2 ^= 0.138, P = 0.013). Additionally, we took into account nonindependence of data using linear mixed-effects model and still observed significant negative regression (Ln(*K*_*n*_*/K*_*s*_) = -0.670 - 0.619×Ln(MED), P = 0.0012).

The densities of plasmid, prophage and transposable element genes separately, also demonstrated significant rank correlations with *K*_*n*_*/K*_*s *_(data not shown). The mobile elements density determined as the number of mobile genes divided by the number of all genes in the genome also demonstrated negative relationships with *K*_*n*_*/K*_*s *_(Kendall rank correlation: tau = -0.329, P < 0.001, N = 46; ordinary linear regression: Ln(*K*_*n*_*/K*_*s*_) = -3.891 - 0.396×Ln(MED), P = 0.008, N = 44).

## Discussion

Unexpectedly, mitochondrial genes, despite low effective population sizes and no recombination, seem to eliminate slightly deleterious mutations more effectively than orthologous genes in obligate intracellular and even free-living bacteria which have huge population sizes and nonzero recombination level.

Moreover, we demonstrated that this result cannot be explained by the positive selection events in bacteria. The *K*_*n*_*/K*_*s *_values were < 1 in all branch-specific models (2a, 2b, 2c), where the *K*_*n*_*/K*_*s *_values were estimated separately for some branches or sub-trees (but were averaged across all sites). Further, the *K*_*n*_*/K*_*s *_values were < 1 for all classes of sites analyzed under site-specific models (7, 8) which account for heterogeneous selective pressure on codons (but are averaged across all branches of the phylogeny). However as the adaptive evolution could affect only few sites at several time points, both approaches of averaging rates over sites or over time (over branches) could fail to detect positive selection. Here we argue that the design of our experiment, especially (I) analysis of conserved genes on (II) external branches of the tree (see model 2c), allows us to disregard the effect of possible positive selection. Indeed, since we analyzed only external branches of the tree, possible events of positive selection in the deep-branch phylogeny did not influence our results. On the other hand it is unlikely that events of positive selection on external branches (for example in bacterial species) could be so numerous and so universal as to fully determine the observed trend.

Our result is unlikely to be biased due to the saturation of synonymous substitutions on long branches or to stochastic errors on too short branches, the since analysis of species with optimal branch length demonstrated similar trends in majority of analyses. Besides, the *K*_*r*_/*K*_*c *_analysis that is less sensitive to the saturation effect, demonstrated similar trends.

Although we used the models that incorporate the possibility of different codon usage between the compared groups of species (see Methods, par. (b)), it is still could be possible, that the codon bias effect was not fully accounted for. We can suppose that free-living bacteria with high Ne possess higher *K*_*n*_*/K*_*s *_values (as compared to mitochondria) due to elimination of some synonymous substitutions through selection for more preferable codons. Such selection would lead to underestimation of *K*_*s*_. To test this possibility we estimated the codon usage as a Kullback-Leibler distance or relative entropy [37]K(f||f′)=∑i=1mfi⋅ln⁡(fifi′)
 MathType@MTEF@5@5@+=feaafiart1ev1aaatCvAUfKttLearuWrP9MDH5MBPbIqV92AaeXatLxBI9gBaebbnrfifHhDYfgasaacH8akY=wiFfYdH8Gipec8Eeeu0xXdbba9frFj0=OqFfea0dXdd9vqai=hGuQ8kuc9pgc9s8qqaq=dirpe0xb9q8qiLsFr0=vr0=vr0dc8meaabaqaciaacaGaaeqabaqabeGadaaakeaacqWGlbWscqGGOaakcqWGMbGzcqGG8baFcqGG8baFcuWGMbGzgaqbaiabcMcaPiabg2da9maaqahabaGaemOzayMaemyAaKMaeyyXICTagiiBaWMaeiOBa4MaeiikaGYaaSaaaeaacqWGMbGzcqWGPbqAaeaacqWGMbGzcuWGPbqAgaqbaaaacqGGPaqkaSqaaiabdMgaPjabg2da9iabigdaXaqaaiabd2gaTbqdcqGHris5aaaa@4C1D@, where *f *is the observed probability distribution of *m *codons in an analyzed genome (downloaded from the Codon Usage Database [38]) and *f' *is the codon frequency under the assumption of uniform synonymous codon usage. If the compared distributions are similar, the relative entropy value should be small. We obtained that the average relative entropy is maximal for genomes of free-living bacteria (mean K = 257, N = 51), intermediate for genomes of obligate bacteria (mean K = 225, N = 19) and minimal for mitochondrial genes (mean K = 191, N = 33). However the comparison of averages did not reveal any significant differences between the groups (the Mann-Whitney U-test) and therefore we concluded that the codon bias should not strongly influence our results.

As we look at only a single sequence from each species and ignore polymorphism within the species, some polymorphic mutations may contribute to *K*_*n*_*/K*_*s *_estimated in our work. For a synonymous substitutions (assuming their neutrality), the expected polymorphism at mutation-drift equilibrium is proportional to the effective population size [2]. At the same time polymorphic slightly-deleterious nonsynonymous substitutions should segregate at a higher frequency in populations with low *N*_*e *_as compared to populations with high *N*_*e *_[2]. Thus the both effects should lead to underestimation of *K*_*n*_*/K*_*s *_values of bacterial genes and overestimation of *K*_*n*_*/K*_*s *_values of mitochondrial genes. Since bacterial *K*_*n*_*/K*_*s *_values, obtained in our work are higher than mitochondrial ones, elimination of the influence of polymorphic substitutions (i.e. increase in bacterial *K*_*n*_*/K*_*s *_values and decrease in mitochondrial *K*_*n*_*/K*_*s *_values) should only strengthen our results.

Thus positive selection, codon usage, stochastic errors or saturation effects and influence of polymorphic substitutions can not provide alternative explanations to the conjecture that purifying selection is more efficient in mammalian mitochondria compared to obligate bacterial species and is at least the same compared to free-living bacterial species.

Most likely, although the mitochondrial genes have the same functions as their bacterial orthologs, they experience stronger evolutionary constraints. There may be several possible causes for that. Firstly, the increased number of subunits in mammalian Complex I (45 [39] versus 14 [40]) can lead to a larger number of protein-protein interactions and consequently to a slower rate of the protein evolution [41]. Secondly, it has been suggested that effective elimination of deleterious mutations from mtDNA during the female germ-cell atresia is caused by the preferential apoptosis of egg cells with defect mtDNA [42-44]. Thirdly, additional factors such as the protein essentiality or the expression level could increase evolutionary constraints on the mitochondrial genes [45,46]. Although it seems likely that both protein essentiality as well as the expression level of the mitochondrial genes should be higher compared to their bacterial counterparts, we are not aware of any experimental studies corroborating these suggestions. Thus future experiments in this area may help to identify the main cause of the increased evolutionary stability of the mitochondrial protein-coding genes as compared to their bacterial orthologs.

The widely accepted opinion about degradation of mitochondrial genome is based on theoretical [47] and empirical backgrounds [48-50]. Since mitochondria possess low effective population size and no recombination, the molecular evolution of mtDNA genes should be associated with a high rate of accumulation of slightly deleterious mutations. A number of studies compared mitochondrial and nuclear genes and corroborated these predictions [48-50]. However, because the genes in nucleus and in mitochondria are not orthologous, this approach is not fully adequate. Here, we compared orthologous genes between mitochondria and various proteobacteria, and observed no degradation of mitochondrial genes. Therefore, we suggest that some other above-mentioned factors have to influence on molecular evolution of mtDNA and maintain highly-effective purifying selection in mitochondria despite their low effective population size and absence of recombination.

Although this study does not refute completely the irreversible accumulation of slightly deleterious mutations in nonrecombining mitochondrial genes, we argue that the rate of the Muller ratchet in modern mammalian mitochondrial genes is at least slower than that in the ecological equivalents of mitochondria, obligate intracellular endosymbionts.

The rate of the Muller ratchet depends on the number of optimal individuals *n*_*o *_with fewest mutations. If *n*_*o *_is small, there is a chance that all n_o _individuals would die without offspring and the ratchet will click round one notch, i.e. the new optimal class of individuals will became slightly worse since it will contain more mutations. Haigh demonstrated that under the additive fitness effect of deleterious mutations, the number of individuals in the optimal class is *n*_*o *_= Ne−U/s
 MathType@MTEF@5@5@+=feaafiart1ev1aaatCvAUfKttLearuWrP9MDH5MBPbIqV92AaeXatLxBI9gBaebbnrfifHhDYfgasaacH8akY=wiFfYdH8Gipec8Eeeu0xXdbba9frFj0=OqFfea0dXdd9vqai=hGuQ8kuc9pgc9s8qqaq=dirpe0xb9q8qiLsFr0=vr0=vr0dc8meaabaqaciaacaGaaeqabaqabeGadaaakeaacqWGobGtdaqhaaWcbaGaemyzaugabaGaeyOeI0IaemyvauLaei4la8Iaem4Camhaaaaa@33C6@, where *N*_*e *_is the effective population size, *U *is the mutational rate per genome per generation, and s is the selection coefficient of deleterious mutations [51]. We argue that n_o _in mitochondria is significantly higher than in obligate intracellular symbionts due to higher s (because of the lower values of *K*_*n*_*/K*_*s *_in mitochondria observed here) and lower or similar *U *(because of the smaller mitochondrial genome size (~16500 bp), but higher mutational rate per nucleotide per generation). Thus, assuming all other parameters equal (*N*_*e *_and mutational rate per nucleotide per generation), the rate of the Muller ratchet is expected to be lower in mitochondria.

Although two of four *K*_*r*_*/K*_*c *_values demonstrate more effective purifying selection in mammalian mitochondria as compared to bacteria, it seems likely, that the *K*_*r*_*/K*_*c *_values in general are less sensitive to the variations in the purifying selection between species.

Higher *K*_*n*_*/K*_*s *_values in obligate intracellular versus free-living bacteria are consistent with the theory as well as with previous observations. This difference is most likely due to low *N*_*e *_and/or recombination level in the obligate intracellular bacteria.

We observed increased effectiveness of purifying selection in obligate intracellular alpha-proteobacterial parasites as compared to obligate intracellular gamma-proteobacterial symbionts. Since both groups pass through the bottlenecks due to their obligate intracellular lifestyle, differences in effective population sizes are unlikely to cause the observed differences in effectiveness of selection. It has been suggested that, since obligate intracellular parasitic microbes change their hosts more frequently than strictly vertically transmitted symbiotic microbes, parasites enjoy recombination among different colonies during horizontal transmissions [22]. Therefore, the most likely explanation for the observed difference in the effectiveness of selection is the higher recombination level in the obligate intracellular parasitic alpha-proteobacteria.

One recently described genome-level difference between obligate intracellular parasites and endosymbionts is the higher number of mobile elements in the former [22]. The negative regression between the mobile element density and the *K*_*n*_*/K*_*s *_values provides empirical evidence that high level of recombination leads to more effective elimination of slightly deleterious mutations. However, causal relationships of various types of mobile elements and purifying selection seem to be different. (I) The negative regression of transposon density on *K*_*n*_*/K*_*s *_is most likely an effect of recombination on both these values, since recombination increases both transposon density and effectiveness of purifying selection (i.e. 1-*K*_*n*_*/K*_*s*_). That would mean that there may be no direct causal links between transposon density and purifying selection. (II) However, other mobile elements, such as plasmids and temperate phages, determine recombination events [23], and therefore their high density causes the increase in the effectiveness of purifying selection. Therefore, recombination induced by mobile elements has an additional role, increasing effectiveness of the purifying selection in bacterial genomes, which is beneficial for the host genome evolution [52,53].

## Conclusion

This study shows that the comparison of patterns of molecular evolution between ecologically different groups elucidates the genetic consequences of various ecological lifestyles. Comparing the strength of the purifying selection among proteobacteria with different lifestyles we obtained results, which are in concordance with theoretical expectations: low effective population size and level of recombination in obligate intracellular proteobacteria lead to less effective elimination of mutations compared to free-living relatives; rare horizontal transmission, i.e. effectively zero recombination level in symbiotic obligate intracellular bacteria leads to less effective purifying selection than in parasitic obligate intracellular bacteria; the high frequency of recombination in bacterial genomes with high mobile element density lead to a more effective elimination of deleterious mutations. At the same time, more effective purifying selection in relatively small populations of asexual mitochondria as compared to large populations of sexual proteobacteria was unexpected since it contradicts the common theory. It seems that additional features such as the high number of protein-protein interactions or female germ-cell atresia maintain the effective purifying selection in mitochondria.

## Methods

### (a) Data and preprocessing

Sequences of seven orthologous genes (mitochondrial: *ND1-6 *and *ND4L*; bacterial: *NuoA*, *NuoH*, *NuoJ*, *NuoK*, *NuoL*, *NuoM*, *NuoN*) of alpha-, beta-, gamma-proteobacteria and mitochondria of primates and glires (*Rodentia *and *Lagomorpha*), were downloaded from the National Center for Biotechnology Information database (NCBI) [54] using blastp [55] with genes of *E. coli K12 *as a query. Thus we obtained the data for 83 bacterial and 34 mammalian species. These genes were then translated into amino acid sequences *in silico*. The obtained amino acid sequences for each gene were aligned using E-INS-i MAFFT model [56,57] and then reverse translated *in silico *to obtain the nucleotide sequence alignments. The aligned amino acid and corresponding nucleotide sequences were concatenated into a single amino acid (nucleotide) sequence for each species (3353 codons in length). The concatenated amino acid sequences were used to reconstruct the phylogenetic trees for each monophyletic group (alpha-, beta-, gamma-proteobacteria, rodents and primates) using PHYML [58,59]. All bacterial trees (for alpha-, beta- and gamma-proteobacteria separately) were rooted using the *Homo sapiens *mitochondrial sequence as an outgroup. Similarly, the primates and glires trees were rooted with *Rickettsia rickettsia *as an outgroup. The constructed mammalian and microbial trees are shown in Figure 1. The homologous codon positions that exist only in bacteria (mammals) and consequently are absent in all mammals (respectively, all bacteria) were removed from all sequences. The resulting sequence was 2126 codons in length. The concatenated nucleotide sequences were used to analyze the pattern of nucleotide substitutions and to reconstruct the nucleotide sequences in the internal nodes of the trees.

### (b) Ratio of nonsynonymous to synonymous nucleotide substitutions (K_n_/K_s_)

The codon-based likelihood model suggested by Goldman and Yang [60] and implemented in the program *codeml *of PAML package [61] provides a useful framework for estimation of synonymous and nonsynonymous substitution rates (*K*_*n*_*/K*_*s*_). The transition/transversion rate bias and the codon frequency bias are found to have significant effects on the estimation of synonymous and nonsynonymous rates, and approximate methods do not adequately account for these factors [62]. Thus, the codon-based likelihood approach is preferable, because this model can easily incorporate these parameters. Because of that, the ratio of the rate of nonsynonymous substitutions to the rate of synonymous substitutions (*K*_*n*_*/K*_*s*_) in this study was estimated using the program *codeml *from the PAML package [61].

To take into account possible species differences in transition/transversion ratio, this parameter was set as free and thus it was estimated from the sequence data in each model. To take into account possible differences in the codon usage bias, all codon frequencies also were set as free parameters.

To estimate the *K*_*n*_*/K*_*s *_values several models were implemented for the mammalian and bacterial trees: model 0 with a single *K*_*n*_*/K*_*s *_value for all branches of the tree; model 2a with two *K*_*n*_*/K*_*s *_values estimated separately for all external and all internal branches; and model 2b with *K*_*n*_*/K*_*s *_values estimated separately for groups of external branches and a single *K*_*n*_*/K*_*s *_value for all internal branches. In model 2b, bacterial external branches were grouped by ecology (two groups: free-living and obligate intracellular organisms) or by taxonomy and ecology (five groups: free-living and obligate intracellular alpha-proteobacteria (parasitic); free-living beta-proteobacteria; free-living and obligate intracellular gamma-proteobacteria (symbiotic)). Since mammalian mitochondria are obligate intracellular symbionts with strictly vertical inheritance, in model 2b the external branches of the mammalian tree were grouped only by taxonomy (primates and glires).

Since an independent estimate of *K*_*n*_*/K*_*s *_for each external branch of the whole tree would have taken too much processor time, the bacterial tree was divided into 3 monophyletic sub-trees (alpha-, beta and gamma-proteobacteria), and the mammalian tree was divided into sub-trees corresponding to glires and primates, and each sub-tree was analyzed separately. For each sub-tree three models were implemented: models 0 and 2a defined as above, and model 2c with *K*_*n*_*/K*_*s *_estimated separately for each external branch and a single *K*_*n*_*/K*_*s *_for all internal branches. The resulting maximum likelihood (ML) values of the models were compared using the likelihood-ratio test [61].

### (c) Positive selection

As only few codons in a gene may be under positive selection, while the rest of the gene is under purifying selection, the average *K*_*n*_*/K*_*s *_value is likely to be below one despite the action of positive selection on the gene. All above mentioned models (see the previous paragraph) assume a single *K*_*n*_*/K*_*s *_ratio for all codons and thus represent a conservative test of positive selection. Adding another class of codons with different *K*_*n*_*/K*_*s *_values may better describe the distribution of *K*_*n*_*/K*_*s *_across codons and reveal codons under positive selection [63,64]. For this goal we used models 7 and 8 in the codeml program. A neutral model (7) with *K*_*n*_*/Ks *values assumed to be beta-distributed on interval (0, 1) was compared with a selection model (8) with one extra class of sites which may have *K*_*n*_*/K*_*s *_values exceeding 1. Positive selection is inferred if the estimate of the *K*_*n*_*/K*_*s *_value of this extra class indeed is larger then one and if the Likelihood Ratio Test (LRT) is significant. The LRT is performed by taking the negative of twice the log-likelihood difference between the nested models (7 and 8) and comparing this to the χ^2 ^distribution with two degrees of freedom.

### (d) Ratio of radical to conservative substitutions (K_r_/K_c_)

Based on the algorithm by Hughes and coauthors [65], which was modified by to take into account the transition/transversion rate bias [66], we computed the rates of conservative (*K*_*c*_) and radical (*K*_*r*_) nonsynonymous substitutions for nucleotide sequences of modern species and their most recent reconstructed ancestors (the ancestral nucleotide sequences were reconstructed using the method by Yang *et al*. [67] implemented in PAML under model 2c). Since the *K*_*c *_and *K*_*r *_rates were small (< 0.3), the Jukes-Cantor formula was used to correct for multiple hits; that is, our *K*_*r*_/*K*_*c *_ratio is identical to the *d*_*R*_/*d*_*C *_ratio in Zhang's notation [66].

The twenty amino acids were classified into groups in four different ways according to their physicochemical properties. We relied largely on Zhang's classification, were the amino acids differ by charge, polarity and both polarity and volume [66]. Additionally, we used Taylor's classification, that classifies the amino acids by volume alone [68]. Amino acid substitutions within groups (i.e. when ancestral and modern amino acids in homologous sites belong to the same group) were regarded as conserved, while those between groups as radical.

### (e) Mobile element genes

For the bacterial species with complete genome sequences available, we determined the number of genes with mobile DNA-related functions. For this we used the automated annotation of the Comprehensive Microbial Resource of the Institute of Genomic Research (TIGR). This annotation classifies genes into nineteen functional-role categories, among which the mobile-DNA category specifies plasmid, prophage and transposable-element genes [69]. We used the number of genes from this category divided by the genome size in megabases as an estimate of the mobile element density in each species.

### (f) Statistical analysis

All statistical analyses were done in the R language (R Development Core Team 2005) [70]. The *K*_*n*_*/K*_*s *_values obtained from models 2a and 2b were compared using the standard error (SE) values from PAML [61]. The *K*_*n*_*/K*_*s *_distributions, obtained using model 2c for pairs of groups with different ecology, were compared using the parametric t-test. The relationship of *K*_*n*_*/K*_*s *_and the mobile elements density was analyzed using three approaches: the Kendall rank correlation which is robust to the nonlinearity of a relationship; the ordinal linear regression for log_e _transformed data; and the regression analysis which takes into account possible non-independence of data. Indeed, since the number of mobile genes may possess some phylogenetic inertia and thus may not be independent among species due to shared ancestry, we performed regression analysis based on the linear mixed-effects model (the function *lme *in the *nlme *package [71] of R language (R Development Core Team 2005)), which explicitly takes into account the hierarchical (nested) structure of comparative-species data [72]. Here, the data are nested into the following two levels: species within families (*Rhizobiales, Rickettsiales; Burkholderiales, Neisseriales; Enterobacteriales, Pseudomonadales*) and within orders (alpha-, beta-, and gamma-proteobacteria, correspondently).

## Authors' contributions

LM found orthologs, mobile elements in bacterial genomes, estimated evolutionary rates and wrote the manuscript. KP performed statistical analyses and edited the manuscript. MSG participated in design and coordination of the study and edited the manuscript. All authors read and approved the final manuscript.

## Supplementary Material

Additional File 1 Average values of *K*_*n *_and *K*_*s *_for each analyzed group. The data provided represent the average values of *Kn *and *Ks *obtained from model 2c for each analyzed group. Click here for file
