# Supplementary material for: Purifying selection in mitochondria, free-living and obligate intracellular proteobacteria
Source: BMC Evol Biol. 2007 Feb 12;7:17. doi: 10.1186/1471-2148-7-17 (PMC1803777; doi:10.1186/1471-2148-7-17)
Supplement: Additional File 1 — Average values of Kn and Ks for each analyzed group. The data provided represent the average values of Kn and Ks obtained from model 2c for each analyzed group. [file 1471-2148-7-17-S1.doc]

Table. Average values of *K_n_* (before slash) and *K_s_* (after slash) for each analysed group. The values are from model 2c.

| mammals  0.0561 / 1.0659 | glires 0.0648 / 1.2080 | |
| --- | --- | --- |
|  | primates 0.0502 / 0.9687 | |
| proteobacteria  0.0350 / 0.4420 | free-living 0.0224 / 0.1906 | beta 0.0065 / 0.1439 |
|  |  | alpha 0.0355 / 0.2115 |
|  |  | gamma 0.0257 / 0.2229 |
|  | obligate 0.0673 / 1.0819 | alpha 0.0363 / 1.3044 |
|  |  | gamma 0.1500 / 0.4884 |
